# Mayaro Virus as the cause of Acute Febrile Illness in the Colombian Amazon Basin

**DOI:** 10.3389/fmicb.2024.1419637

**Published:** 2024-07-09

**Authors:** Laura S. Perez-Restrepo, Karl Ciuoderis, Jaime Usuga, Isabel Moreno, Vanessa Vargas, Angela J. Arévalo-Arbelaez, Michael G. Berg, Gavin A. Cloherty, Juan Pablo Hernández-Ortiz, Jorge E. Osorio

**Affiliations:** ^1^GHI One Health Colombia, Universidad Nacional de Colombia, Medellín, Colombia; ^2^Abbott Pandemic Defense Coalition, Chicago, IL, United States; ^3^Infectious Diseases Research, Abbott Diagnostics, Abbott Park, IL, United States; ^4^Faculty of Life Sciences, Universidad Nacional de Colombia, Medellín, Colombia; ^5^Department of Pathobiological Sciences, School of Veterinary Medicine, University of Wisconsin, Madison, WI, United States; ^6^Global Health Institute, University of Wisconsin, Madison, WI, United States

**Keywords:** Mayaro Virus, Acute Febrile Illness, whole genome sequencing, Colombia, fever

## Abstract

**Introduction:**

Mayaro Fever (MF) is a tropical disease caused by the Mayaro virus (MAYV), with outbreaks documented in Latin America.

**Methods:**

A hospital-based fever surveillance in Leticia, Colombian Amazon, collected sera from 1,460 patients aged 5-89 between December 2020 and April 2023.

**Results:**

Dengue and malaria were the main diagnoses (19.4 and 5.8%, respectively), leaving 71.4% of cases unidentified after testing. Metagenomic sequencing and real-time RT-qPCR testing identified MAYV in two patients (25-year-old male and an 80-year-old female) exhibiting typical symptoms, of MF including rash, joint pain, and fever. Phylogenetics analysis of these two viruses revealed a close relationship to Peruvian strains within the MAYV D genotype.

**Discussion:**

The study of AFI in Leticia, Colombia, identified dengue as prevalent, with malaria, COVID-19, Influenza, and Zika viruses also detected. Despite extensive testing, most cases remained unexplained until metagenomic sequencing revealed MAYV, previously unseen in Colombia but known in neighboring countries.

**Conclusion:**

This study presents the first near full-length genomes of MAYV in Colombia, highlighting the need for further seroprevalence studies and enhanced surveillance to understand and control the spread of the virus in the region.

## Introduction

Mayaro Virus (MAYV) is an arthropod-borne, single-stranded RNA virus that belongs to the Semliki Forest antigenic sero-complex, a serological group within the alphavirus genus (family *Togaviridae*) (Acosta-Ampudia et al., 2018). MAYV was initially identified in the Mayaro county, Trinidad in 1954 and since then, several cases of Mayaro fever (MF) have been reported in Latin America and the Caribbean (Caicedo et al., 2023). Four distinct MAYV genotypes have been recently identified in South America (Mavian et al., 2017). MAYV infections pose a significant health concern in Latin America, particularly in regions like South America, where the virus is endemic. Brazil, Peru, and Venezuela have documented the highest incidence and prevalence of Mayaro fever (Del Carpio-Orantes et al., 2022). Despite being relatively understudied compared to other mosquito-borne viruses like dengue and Zika, MAYV has been identified as a cause of Acute Febrile Illness (AFI) in the region. While cases vary due to underreporting and limited surveillance, sporadic outbreaks and localized transmission have been documented, particularly in areas with favorable ecological conditions for the virus and its mosquito vectors (Diagne et al., 2020). The impact of MAYV infections extends beyond the immediate health effects, often affecting vulnerable populations and burdening healthcare systems already strained by other infectious diseases. Understanding the epidemiology and impact of MAYV infections is crucial for effective public health responses and mitigating the spread of the virus in Latin America.

Acute Febrile Illness (AFI) represents a significant health challenge in tropical regions, where many infectious pathogens circulate, often leading to similar clinical presentations. Clinical manifestations may include arthralgia/arthritis, a maculopapular rash, and other symptoms such as headache, myalgia, retro-orbital pain, vomiting, and diarrhea (Acosta-Ampudia et al., 2018). Diagnosing MF can be difficult because its signs and symptoms can be easily confused with other co-occurring infections such as malaria and arboviral diseases such as dengue, chikungunya, and Zika (Arroyave et al., 2013). Consequently, confirmatory laboratory testing is required (Diagne et al., 2020). However, it is very limited in most of the tropical areas. Despite extensive laboratory screening, many AFI cases remain without a specific etiology identified. This diagnostic gap hampers individual patient management and impedes effective public health responses to emerging infectious threats (Barathan, 2024). Metagenomic next-generation sequencing (mNGS), a powerful molecular technique capable of detecting a broad range of pathogens directly from clinical samples, offers a promising solution to this diagnostic dilemma (Batool and Galloway-Peña, 2023). Metagenomic sequencing can unveil the presence of known and novel pathogens by analyzing the entire genetic content within a sample, providing crucial insights into disease causation and facilitating targeted interventions for diagnosed and undiagnosed AFI cases in tropical regions. Several studies on AFI in Colombia have observed a great proportion of febrile cases that remained with unknown diagnoses after routine testing or disease investigation. Therefore, this study aimed to employ mNGS as a diagnostic tool to identify the causative agents of AFI in different regions of Colombia where traditional laboratory methods often fail to provide etiological diagnoses. Through comprehensive genomic analysis of clinical samples, we aimed to elucidate the spectrum of pathogens contributing to AFI, including known and novel infectious agents. By characterizing the microbial diversity in these samples, our goal was to enhance our understanding of AFI epidemiology, inform clinical management strategies, and contribute to developing targeted interventions for improved public health outcomes in tropical settings such as Colombia.

## Materials and methods

### Study setting

Repository samples used in this study were obtained from a cross-sectional hospital-based fever surveillance program (HFSP) conducted since December 2020 in four different regions of Colombia (Villavicencio, Apartado, Acacias, and Leticia; Figure 2A). This program is part of an ongoing Virus Discovery Research Plan of the Abbott Pandemic Defense Coalition (Averhoff et al., 2022) aimed at understanding infectious causes causing AFI of unknown origin. This study was reviewed and approved by the Ethics Committee of Corporacion Investigaciones Biologicas (CIB 10102022). Written informed consent was secured from adults (18 years or older). Informed assent was obtained from minors (<18 years), and written consent was obtained from their parents or guardians on their behalf. At the enrollment, participants agreed to use their data and left-over specimens for future studies. For the purpose of this study, acute-phase sera collected (from December 2020 to April 2023) from 1,460 febrile individuals (aged 5–89 years) from the municipality of Leticia (located in the Amazon region of Colombia) were used.

**FIGURE 1:**
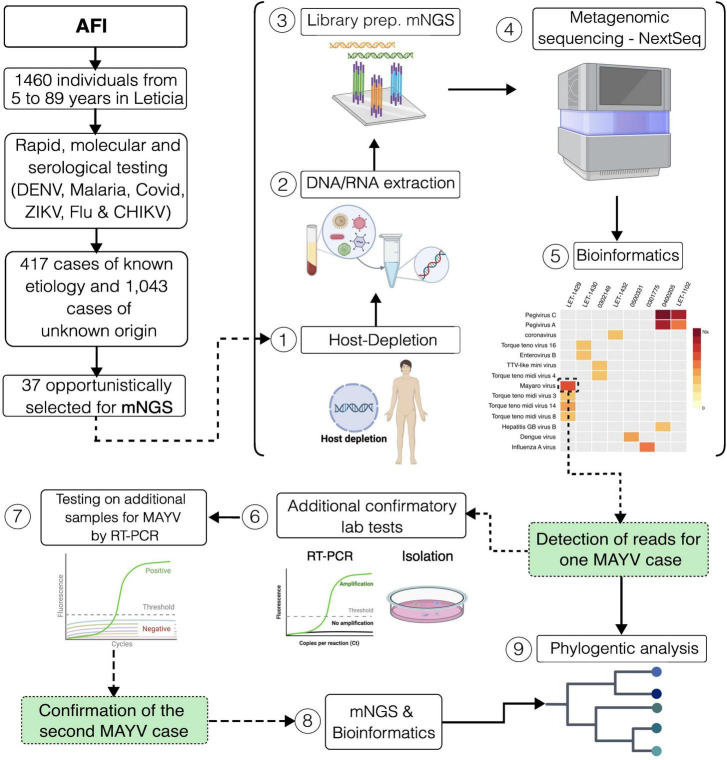
Graphical summary of the study design. Study setting and workflow for sample processing.

**FIGURE 2:**
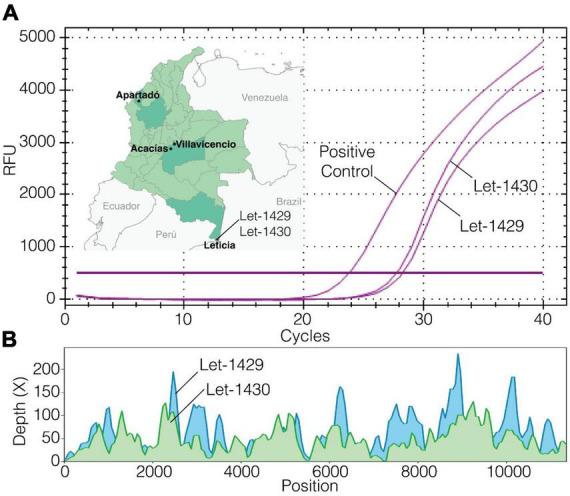
Fever surveillance, Mayaro virus (MAYV; Let-1429 and Let-1430) detection, viral isolation, and sequencing. **(A)** Map of Colombia showing the location where the Virus Discovery Research Program is conducted and origin of MAYV infections detected, and real-time RT-PCR test results on cell culture supernatants after 72 h of infection; **(B)** mNGS coverage plot for near full virus genomes.

### AFI case definition

For this study, AFI was defined as recent onset of fever (body temperature ≥ 38°C at the time of consultation or self-reported history of fever within the preceding seven days) without an obvious focus of infection. AFI was associated with non-specific symptoms such as headache, body rash, and muscle and joint pains (Tun et al., 2016).

### Laboratory testing

Samples were initially tested for dengue, malaria at the point-of-care using rapid diagnostic tests (SD Bioline Dengue Duo and Bioline Malaria Ag P.f/Pan, Abbott, IL, USA). Serum and whole blood samples were aliquoted and stored at −80°C until processing at the central lab (One Health Genomic Lab, Universidad Nacional de Colombia, Medellin). Samples were tested by molecular assays (reverse transcription RT-PCR and/or PCR) to detect Malaria, Dengue (DENV), Zika (ZIKV), Chikungunya (CHIKV), Influenza (IV), and Severe-Acute-Respiratory-Syndrome-related Coronavirus (SARS-CoV-2), following protocols described elsewhere (de-Paris et al., 2012; Kamau et al., 2013; Waggoner et al., 2016; Corman et al., 2020). All samples with unknown etiology were kept at −80°C for further testing.

### Virus discovery and sequencing

After laboratory testing, a subset (*n* = 37) of the repository specimens with unknown etiology was non-randomly selected based on the reported clinical manifestations such as vomit, abdominal pain, skin rash, hemorrhages, and/or diarrhea. Selected samples were subjected to Metagenomic Next Generation Sequencing (mNGS). mNGS was conducted using a NextSeq2000 (Illumina, California, USA) following a protocol described elsewhere (Berg et al., 2020). Contamination was identified when pathogens detected by mNGS did not align with the clinical diagnosis, had not been clinically confirmed, and were not regarded as the cause of the clinical disease. Sequencing data were analyzed using an open-source cloud-based metagenomics platform (Tun et al., 2016; Kalantar et al., 2020) using the following filters: category (viruses and viroids), subcategory (Viruses – Phage), threshold (NT rPM > = 10; NR rPM > = 5; NR L (alignment length in bp) > = 50), and pathogen tag (known_pathogens).

### Bioinformatics and phylogeographic analysis

Sequence alignment was performed on the MAYV sequences from this study using Samtools v1.15 and BWA v0.7.17 (Li and Durbin, 2009; Li et al., 2009). Complete genome sequences of MAYV were downloaded from the public database GenBank (National Center for Biotechnology Information) and compared to the sequences from this work using MAFFT v7.520 (Katoh et al., 2019). A Bayesian phylogeographic analysis was performed (Figure 2) on the MAYV sequences obtained as described previously (Ciuoderis et al., 2023).

## Results

From December 2020 to April 2023, acute-phase sera were collected from 1,460 febrile individuals (aged 5–89 years, and 57.7% were female) from the municipality of Leticia, located in the Amazon region of Colombia. A total of 19.4% (284/1460) patients were confirmed as dengue after laboratory testing, while 5.8% (85/1460) were diagnosed as malaria, 2.9% (43/1460) as COVID-19, 0.3% (4/1460) as Influenza, and 0.1% (1/1460) as Zika, respectively (Table 1). However, 71.4% (1043 out of 1460) of these AFI cases remained unexplained (Figure 1). To address this, 37 samples were non-randomly selected for mNGS (Figure 1). Most confirmed dengue cases occurred in individuals aged 11–35 years, with peak incidences occurring during the rainy season (second and third trimester). Malaria cases were predominantly found in males aged above 35 years, with peak incidences occurring during July and September. COVID-19 cases were predominantly found in females aged 26–35 years, with incidences occurring throughout the period of study. Influenza cases were widely distributed among age groups and throughout the study period.

**TABLE 1:** Diagnostics test results on serum samples from febrile patients in Leticia, Colombia.

Diagnostic test	Results (%)	Reference method
DENV RT-PCR	284/1460 (19.4)	Waggoner et al., 2016
DENV Duo RDT	263/1460 (18.0)	Bioline Dengue Duo (Dengue NS1 Ag + IgG/IgM), Abbott, Illinois, USA
	309/1460 (21.2)	
	973/1460 (66.6)	
Malaria PCR	85/1460 (5.8)	Kamau et al., 2013
Malaria RDT	83/1460 (5.7)	SD Bioline Malaria Ag P.f/Pan, Abbott, Illinois, USA
SARS-CoV-2 RT-PCR	43/1460 (2.9)	Corman et al., 2020
Influenza RT-PCR	4/1460 (0.3)	de-Paris et al., 2012
ZIKA RT-PCR	1/1460 (0.1)	Waggoner et al., 2016
MAYV RT-PCR	1/80 (1.25)	Waggoner et al., 2016
Negative to all laboratory tests	1043/1460 (71.4)	

RT-PCR, reverse transcription polymerase chain reaction; RDT, rapid diagnostic testing.

Results from mNGS revealed that a near full-length (94%) genome of MAYV was detected in one specimen (Figure 2B). This MAYV infection was referred to as the index case, occurring on April 2023 in a 25-year-old male (Let-1430) of Indigenous ethnicity. The subject reported fever, rash, joint pain, retro-orbital pain, shaking chills, abdominal pain, weakness, myalgia, headache, vomiting, and diarrhea. The subject did not report recent travel to areas outside of the vicinity. Additional serum specimens of unknown etiology (*n* = 80) from Leticia were tested for MAYV infection by real-time RT-qPCR, targeting the untranslated region of the nonstructural protein 1 gene of MAYV. The RT-qPCR method was conducted following a protocol described elsewhere (Waggoner et al., 2018). Serum samples were selected within a month before and after the index case was identified, and from these, another MAYV infection case was detected. This case occurred in an 80-year-old woman of mixed race (Let-1429) who reported the same symptoms as the index case except for the rash. Metagenomic NGS was also conducted in this specimen, identifying reads for MAYV with high identity (>95%) to the reference genome NC_003417. MAYV isolates were successfully obtained from both cases after inoculating infected serum samples onto sub-confluent Vero cells, following a protocol described elsewhere (Blohm et al., 2019). Virus isolation was confirmed by RT-qPCR (Figure 2A).

**FIGURE 3:**
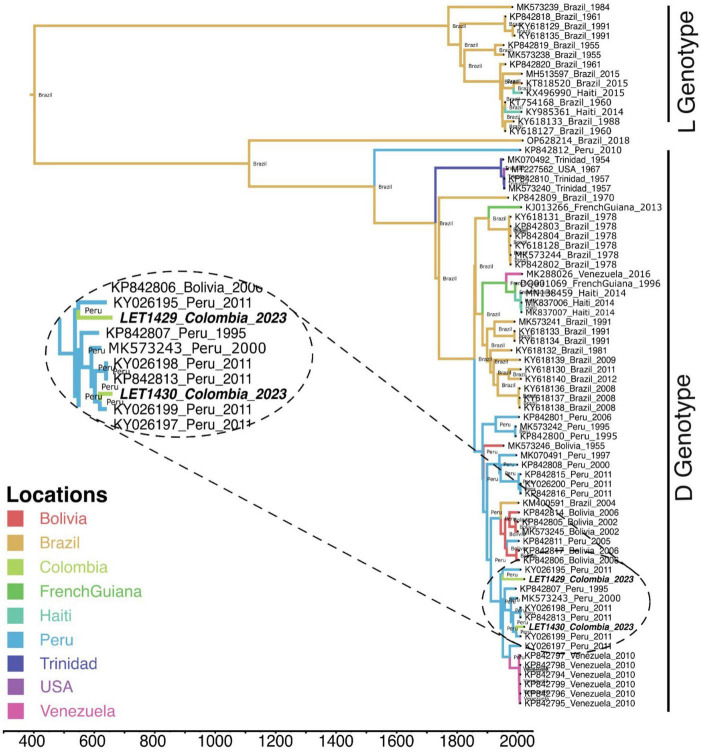
Phylogeographic and phylogenetic analysis of Mayaro virus from Colombia. A time-scaled phylogeographic maximum clade credibility tree was constructed using Bayesian Markov Chain Monte Carlo simulations with Beast v1.10.4. The model GTR + I + G was selected as the best-fit model based on Bayesian information criteria using ModelFinder from IQ-Tree program v2.1.2. The analysis included MAYV sequences from this study (bold italic) along with sequences available from GenBank. Branch colors indicate the phylogeographic origin. Bayesian posterior values (≥0.7) are shown in nodes.

After mNGS, phylogenetic analysis revealed that MAYV from this study clustered together with genotype D sequences reported from Peru. In addition, phylogeographic analysis using available MAYV genomes revealed that MAYV strains in Colombia may have been introduced from Peru through two independent events (Figure 3) and, therefore, have not originated from a single outbreak. Two full-length genomes of MAYV analyzed in this work were deposited in the GenBank under accession numbers PP505831 and PP505832. Genomic sequencing data analysis for other samples revealed Pegivirus C (*n* = 1), *Prevotella melaninogenica* (*n* = 11), *Aeromona caviae* (*n* = 9), *Mycobacteroides chelonae* (*n* = 6), *Haemophilus influenzae* (*n* = 3), *Staphylococcus lugdunensis* (*n* = 2), *Mycobacterium avium* (*n* = 2), *Klebsiella aerogenes* (*n* = 1), *Bordetella bronchiseptica* (*n* = 1) as the most common atypical pathogens identified by mNGS.

## Discussion

After investigating the landscape of AFI among a cohort of 1,460 individuals in the municipality of Leticia, situated within the Amazon region of Colombia, and primarily focusing on the identification and characterization of pathogens causing such illness, we found that a multifaceted picture emerged with the highest number of cases confirmed of dengue and smaller occurrences of malaria, COVID-19, Influenza and Zika. However, most (71.4%) of AFI cases remained unexplained. MNGS and other laboratory techniques were employed within this enigmatic pool, leading to the pivotal discovery and characterization of MAYV. To our knowledge, while MAYV is recognized as a source of AFI in South America, there have been no documented cases of MAYV infections in Colombia to date.

In agreement with our findings, genotype D of MAYV has been the most reported circulating genotype in South America (Mavian et al., 2017). Furthermore, there is strong evidence that MAYV is the cause of AFI outbreaks in neighboring countries, central and South America (Lorenz et al., 2019; Celone et al., 2023). A recent report from Peru showed that MAYV infections occurred in 17.3% of 496 febrile cases studied, of which 10.9% were MAYV mono-infections and 6.4% were co-infections with DENV (Aguilar-Luis et al., 2021). Similarly, another study in the same country confirmed MAYV infection by RT-PCR and sequencing in 11.1% (40/359) of the febrile patients tested (Aguilar-Luis et al., 2020). Confirmation of MAYV infections has recently been reported in mosquitoes and humans in Brazil (Saatkamp et al., 2021; de Curcio et al., 2022). Natural MAYV infection has also been documented in Culicidae mosquito species in several South American countries, including Colombia (Caicedo et al., 2023), but additional evidence supporting their involvement in the MAYV transmission in Colombia is required.

There is some evidence of natural infection of MAYV in vector mosquitoes (*Psorophora albipes*) in the northeast region of Colombia (Caicedo et al., 2023). However, to our knowledge, MAYV infection in humans has not been previously reported in this country. In addition, detection of MAYV infection may also be challenging when using only molecular testing. The relatively short (approximately 3–10 days) viremia could limit MAYV detection (Pezzi et al., 2019). Therefore, further seroprevalence studies are highly recommended to comprehensively assess the burden of this disease in the Amazon River basin and Colombia. A recent review of population-based studies reported a seroprevalence of MAYV infection ranging between 6 and 67% for South American countries (Caicedo et al., 2023), and recently, a seroprevalence study in Mexico showed that MAYV infection occurred in 1% of adults who reported having suffered from an arboviral illness at some point in their lives (Del Carpio-Orantes et al., 2022). Consequently, our findings indicate that MAYV infections may be cryptically occurring in some regions of Colombia, and cases are being misdiagnosed, thus highlighting the need for active surveillance of MAYV in Colombia.

Atypical viral pathogens such as Pegivirus C and bacteria such as *Klebsiella pneumoniae* and non-tuberculous mycobacteria have been reported in febrile and patients with respiratory illness after metagenomic sequencing analysis (del Valle-Mendoza et al., 2017; Oguzie et al., 2023; Shen et al., 2023; Wei et al., 2023; Yang et al., 2024). However, further research is needed to assess its implications in disease pathogenesis, specifically in disease cases of AFI of unknown origin.

Our results contribute to the understanding of MAYV in the Americas and emphasize the importance of implementing metagenomic NGS to detect pathogens causing AFI of unknown origin, especially when other specific detection methods, such as PCR, are not available routinely used for diagnosis. There is also a need to develop commercially available diagnostic tests for MAYV to understand the disease burden better. Finally, we strongly suggest increasing educational campaigns about MAYV for the community, especially for healthcare personnel in high-risk areas of Colombia. Such targeted strategies are pivotal to ensure that communities are aware of this disease and that healthcare professionals in the region are provided with the most recent knowledge to proficiently detect, manage, and address these MAYV infections.

## Data availability statement

The datasets presented in this study can be found in online repositories. The names of the repository/repositories and accession number(s) can be found here: https://www.ncbi.nlm.nih.gov/genbank/, PP505831, https://www.ncbi.nlm.nih.gov/genbank/, PP505832.

## Ethics statement

The studies involving humans were approved by the Ethics Committee of Corporación de Investigaciones Biológicas. The studies were conducted in accordance with the local legislation and institutional requirements. Written informed consent for participation in this study was provided by the participants’ legal guardians/next of kin.

## Author contributions

LP-R: Conceptualization, Data curation, Formal analysis, Methodology, Writing – original draft, Writing – review and editing. KC: Conceptualization, Data curation, Formal analysis, Methodology, Writing – original draft, Writing – review and editing. JU: Data curation, Methodology, Writing – review and editing. IM: Methodology, Writing – review and editing. VV: Methodology, Writing – review and editing. AA-A: Methodology, Writing – review and editing. MB: Formal analysis, Resources, Writing – review and editing. GC: Funding acquisition, Supervision, Writing – review and editing. JH-O: Writing – review and editing. JO: Funding acquisition, Supervision, Writing – review and editing.
